# Breathing-Based Meditation Decreases Posttraumatic Stress Disorder Symptoms in U.S. Military Veterans: A Randomized Controlled Longitudinal Study

**DOI:** 10.1002/jts.21936

**Published:** 2014-08-26

**Authors:** Emma M Seppälä, Jack B Nitschke, Dana L Tudorascu, Andrea Hayes, Michael R Goldstein, Dong T H Nguyen, David Perlman, Richard J Davidson

**Affiliations:** 1 Center for Compassion and Altruism Research and Education, School of Medicine, Stanford UniversityStanford, California, USA; 2 Department of Psychology, University of Wisconsin-MadisonMadison, Wisconsin, USA; 3 Department of Psychiatry, University of Wisconsin-MadisonMadison, Wisconsin, USA; 4 Department of Internal Medicine, Biostatistics and Geriatric Psychiatry Neuroimaging Lab, University of PittsburghPittsburgh, Pennsylvania, USA; 5 Waisman Laboratory for Brain Imaging and Behavior, University of Wisconsin-MadisonMadison, Wisconsin, USA; 6 Department of Psychology, University of ArizonaTucson, Arizona, USA

## Abstract

Given the limited success of conventional treatments for veterans with posttraumatic stress disorder (PTSD), investigations of alternative approaches are warranted. We examined the effects of a breathing-based meditation intervention, Sudarshan Kriya yoga, on PTSD outcome variables in U.S. male veterans of the Iraq or Afghanistan war. We randomly assigned 21 veterans to an active (*n* = 11) or waitlist control (*n* = 10) group. Laboratory measures of eye-blink startle and respiration rate were obtained before and after the intervention, as were self-report symptom measures; the latter were also obtained 1 month and 1 year later. The active group showed reductions in PTSD scores, *d* = 1.16, 95% CI [0.20, 2.04], anxiety symptoms, and respiration rate, but the control group did not. Reductions in startle correlated with reductions in hyperarousal symptoms immediately postintervention (*r* =. 93, *p* <. 001) and at 1-year follow-up (*r* =. 77, *p* =. 025). This longitudinal intervention study suggests there may be clinical utility for Sudarshan Kriya yoga for PTSD.

Many veterans from the Operation Enduring Freedom (Afghanistan war) or Operation Iraqi Freedom (Iraq war) suffer from pronounced posttraumatic stress disorder (PTSD) symptoms that contribute to alarming suicide rates (Panagioti, Gooding, & Tarrier, 2009; Sher, Braquehais, & Casas, 2012; U.S. Department of Veteran Affairs, 2012). Despite promising advances in evidence-based treatments for PTSD (Cukor, Olden, Lee, & Difede, 2010), dropout rates remain as high as 54.0% in populations with PTSD (Brown, 2012; Schottenbauer, Glass, Arnkoff, Tendick, & Gray, 2008) and up to 62.4% in Afghanistan or Iraq veterans (e.g., Harpaz-Rotem & Rosenheck, 2011). Furthermore, results of pharmaceutical treatments are mixed (Alderman, McCarthy, & Marwood, 2009), whereas substantial residual symptoms remain after psychotherapy (Bradley, Greene, Russ, Dutra, & Westen, 2005), suggesting a critical need to evaluate alternative or supplementary approaches to treating PTSD.

A growing body of evidence suggests meditation-based interventions have the potential to reduce symptoms and improve well-being (see Marchand, 2013 for review; Mitchell et al., 2014). The use of meditation, however, as an intervention for PTSD in veterans has not been sufficiently studied (Lang et al., 2012; U.S. Department of Veteran Affairs, 2011). Breathing-based meditation practices may be particularly beneficial for PTSD given that it is a stress disorder characterized by hyperarousal, autonomic dysfunction, negative affect, and difficulties with emotion regulation (American Psychiatric Association, 2013; Sack, Hopper, & Lamprecht, 2004). Respiration and emotion are tightly coupled processes with bidirectional influence (Boiten, Frijda, & Wientjes, 1994; Philippot, Chapelle, & Blairy, 2002) and breathing interventions have boosted emotion regulatory processes in healthy populations (Arch & Craske, 2006). They have also normalized parasympathetic activity in anxious populations not suffering from PTSD (Asmundson & Stein, 1994; Kaushik, Kaushik, Mahajan, & Rajesh, 2006; Salkovskis, Jones, & Clark, 1986) and in healthy populations with experimentally induced anxiety (Sakakibara & Hayano, 1996).

Therefore, we investigated the impact of a breathing-based meditation, Sudarshan Kriya yoga, in Afghanistan or Iraq veterans with PTSD symptoms. We selected Sudarshan Kriya yoga because it has effectively reduced PTSD symptoms in tsunami survivors (Descilo et al., 2009), increased self-reported optimism and well-being in college students (Kjellgren, Bood, Axelsson, Norlander, & Saatcioglu, 2007), decreased self-reported anxiety in those with general anxiety disorder (Katzman et al., 2012), and decreased self-reported depression in those with melancholic depression (Janakiramaiah et al., 2000) as well as in alcohol-dependent inpatients (Vedamurthachar et al., 2006). Studies have shown that it improves emotion regulation in yoga practitioners (Gootjes, Franken, & Van Strien, 2011), reduces impulsive behavior in adolescents (Ghahremani, Oh, Dean, Mouzakis, Wilson, & London, 2013), curbs tobacco consumption and improves immune function in cancer patients (Kochupillai et al., 2005), lowers blood lactate and increases antioxidant levels in Sudarshan Kriya practitioners (Sharma et al., 2003), and increases rapid gene expression in a non-clinical population (Qu, Olafsrud, Meza-Zepeda, & Saatcioglu, 2013).

The present study used a multimethod approach—self-report symptom measures and psychophysiological testing—to assess the effects of Sudarshan Kriya yoga. We hypothesized there would be reductions in PTSD, anxiety, and physiological startle response. Given the use of a respiration-based meditation and data indicating that similar interventions slow respiration rate in anxious populations (e.g., Delgado et al., 2010; Kaushik et al., 2006), we also measured respiration rate at baseline and one week after, well as following the intervention at two additional follow-up intervals.

## Method

## Participants

Participants learned about the study through flyers and veteran and military organizations and contacted the laboratory by telephone for prescreening. Those who met selection criteria were invited for an in-person screening. Participants provided written informed consent for the protocol approved by the University of Wisconsin-Madison Institutional Review Board.

**Figure 1:**
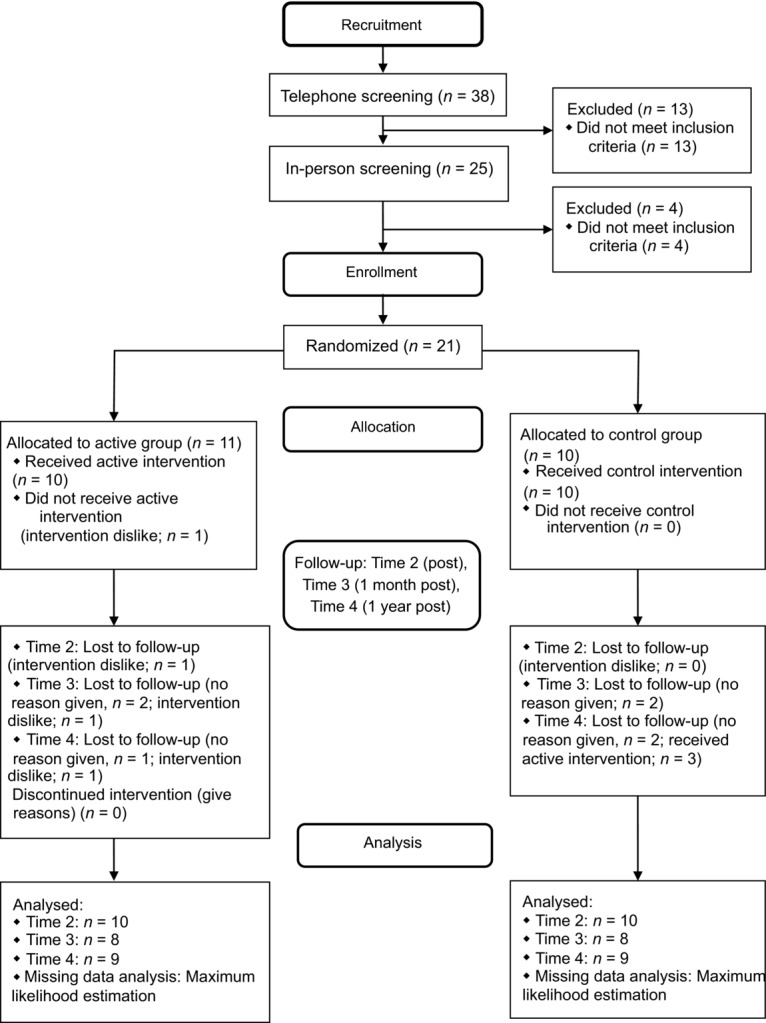
Diagram summarizing participant flow for recruitment, enrollment, allocation, follow-ups, and analysis.

Inclusion criteria were male gender (to control for effects of sex), 18 years of age or older, English fluency, and veteran with service in Afghanistan or Iraq. Participants were excluded if they reported substance dependence, psychosis, or use of alpha- or beta-blocking medications because of possible interference with psychophysiological measures. There were 38 veterans who made contact, 25 were screened in person, and 21 met eligibility criteria (Figure 1).

Demographics were collected prior to group assignment to assess inclusion criteria as part of our standard intake procedure. A biostatistician determined the simple randomization procedure using a computer-generated randomization list. The study coordinator then assigned eligible participants to the groups according to the randomization list: Sudarshan Kriya yoga active (*n* = 11) or waitlist control (*n* = 10) group. All primary outcome measures were administered postrandomization. The randomization procedure proved effective: The groups were similar for age (active: *M* = 28.09 years, *SD* = 2.91; control: *M* = 29.20, *SD* = 6.66), ethnicity (active: nine Caucasian, two Other; control: nine Caucasian, one Other), marital status (active: four married, seven not married or divorced; control: four married, six not married or divorced), education (active: eight with less than 4-year college degree, three with a 4-year college degree or higher; control: seven with less than a 4-year college degree, three with a 4-year college degree or higher), and military service length (active: *M* = 7.05 years, *SD* = 2.60; control: *M* = 9.26, *SD* = 7.29). The groups were also similar for combat exposure (active: *M* = 17.91, *SD* = 6.47; control: *M* = 16.70, *SD* = 7.65) as measured by the Combat Exposure Scale (Keane et al., 1989), which evaluates the nature and severity of exposure to traumatic combat experiences. For overall PTSD ratings via the PTSD Checklist-Military scale (Blanchard, Jones-Alexander, Buckley, & Forneris, 1996; Bliese et al., 2008), the active and control groups had similar scores (active: *M* = 36.55, *SD* = 11.44; control: *M* = 32.40, *SD* = 13.34) comparable with suggested cutpoints of 30–35 (U.S. Department of Veteran Affairs National Center for PTSD, 2012). We did not formally assess clinical comorbidities aside from the self-reported measures described in the Procedure section. Two out of 10 participants in the active group and one out of 10 participants in the control group had been in therapeutic treatment for at least 3 months prior to the study. One participant in the active group and one participant in the control group were taking antianxiety and antidepressant medication. We recommended participants continue current treatment.

## Procedure

All subjective and objective laboratory assessments for the active group were conducted within 1 week before (Time 1) and 1 week after (Time 2) the 7-day intervention. To control for season and time effects, the control group underwent laboratory assessments during the same month (November 2010). Long-term efficacy was assessed via online self-report questionnaires 1 month (Time 3) and 1 year (Time 4) postintervention.

All 21 participants completed physiological and self-report assessments at Time 1. One participant in the active group dropped out after the third day because he disliked the intervention. Ten participants in each group completed assessments at Time 2. Eight from each group completed assessments at Time 3 (1-month postintervention), whereas nine participants in the active group and eight in the control group completed assessments at Time 4 (1-year postintervention). Three of eight control participants received the Sudarshan Kriya yoga intervention between Times 3 and 4; therefore, their Time 4 data were excluded. No harm or unintended effects were reported for either group.

Sudarshan Kriya yoga is a group-oriented, manual-based, controlled breathing meditation intervention that focuses on several types of breathing exercises with periods of discussion and stretching. The exercises include four sequential, form- and rhythm-specific breathing components interspersed with normal breathing while sitting with eyes closed (see Brown and Gerbarg, 2005a for details). During the 7-day, 21-hour intervention, participants met daily for 3 hour-long group sessions. Instructors were from *Project Welcome Home Troops* (http://www.pwht.org), a project of the International Association of Human Values (http://www.iahv.org).

Using a published veteran-specific startle paradigm (Grillon, Morgan, Davis, & Southwick, 1998), we recorded eye-blink responses to 24 acoustic startle probes (50 millisecond [ms], 107 decibel white noise bursts with near-instantaneous rise and fall times administered through earbuds and presented at 18–25-second [s] intervals) with illumination of the laboratory room switching between light and dark every four probes (no probes for the first 18–25 s of each light/dark period). The change in illumination was intended to elicit a higher startle response in the dark (Grillon et al., 1998). Electromyography was recorded from the orbicularis oculi muscles using silver-silver chloride EL254 BIOPAC electrodes (BIOPAC Systems, Inc., Goleta, CA) placed directly below and lateral to the left or right eye (impedances < 20 kilohm [kΩ]). Startle reflex magnitude was computed as the difference between peak amplitude (20–120 ms after probe) and onset amplitude (20–60 ms after probe). Blinks greater than 3 *SD*s above each participant's mean were excluded.

We recorded respiration using a BIOPAC respiration belt that was connected to a BIOPAC Systems RSP100C amplifier and placed around participants’ chest below the sternum. Respirations were counted throughout the 5-minute [min] baseline period. The data were divided into 50-s windows and two blind independent raters manually counted respiration cycles per window. Windows with rater disagreements were excluded from analyses (23 of 400 windows) and valid windows were averaged across the 5-min baseline.

## Measures

PTSD was assessed with the PTSD Checklist-Military version (PCL-M; Blanchard et al., 1996), which comprises three subscales–Reexperiencing, Avoidance, and Hyperarousal. The Mood and Anxiety Symptoms Questionnaire (MASQ; Watson et al., 1995) assessed general and specific components of anxiety and depression via four subscales: General Distress-Anxiety, Anxious Arousal, General Distress-Depressive, and Anhedonic Depression. Internal consistency of all subscales and total scores at each of the four time points was moderate to high (PCL-M: Cronbach's α =. 76 to. 95; MASQ: α =. 70 to. 97).

## Data Analysis

Primary outcome measures were self-reported PTSD scores. Secondary outcome measures were self-reported anxiety and depression, psychophysiological measures of startle response, and respiration rates.

To address the issue of missing and unusable data, we implemented an intent-to-treat analysis using the maximum likelihood estimation. A mixed models approach is optimal for small data sets because it uses all available data without eliminating participants with incomplete cases (Littell, Milliken, Stroup, Wolfinger, & Schabenberger, 2006; Pinheiro & Bates, 2000). Linear mixed-effects models were used for all variables to test main effects of time (Time 1, Time 2, Time 3, Time 4), group (control, active), and group-by-time interactions as well as to account for within-subjects correlation. One between-subjects factor (group) and one within-subjects factor (time) and their interaction were defined as fixed effects. Subject was defined as a random effect.

After review of the correlation within subjects, a compound symmetry structure was assumed for the self-report measures. Correlations were conducted to test the anticipated relationship between physiological startle, respiration, and PTSD symptoms. To estimate the magnitude of the active group's intervention effects relative to Time 1 above and beyond changes in the control group, between-group Cohen's *d* effect sizes were calculated using the difference scores per group (Cohen, 1992). Analyses were conducted using two-tailed tests in SAS (SAS Institute Inc., SAS Campus drive, Cary, NC), SPSS 20.0, and STATISTICA 10 (StatSoft Inc., Tulsa, OK).

## Results

The absence of group differences at Time 1 for all measures (all *p*s >. 108) indicated that the randomization procedure for group assignment was successful.

Regarding PTSD symptoms, a Group × Time interaction was observed for the PCL–M, *F*(3, 44.92) = 4.52, *p* =. 007. The active group showed fewer symptoms at Time 2, *t*(45.08) = 4.39, *p* <. 001, Time 3, *t*(45.22) = 3.17, *p* =. 003, and Time 4, *t*(45.15) = 4.54, *p* <. 001, compared to Time 1 (Table 1).

**Table 1:** Means and Standard Deviations at Four Time Point for Two Groups

	Time Control							
	1 (*n* = 10)		2 (*n* = 10)		3 (*n* = 8)		4 (*n* = 5)	
Measure	*M*	*SD*	*M*	*SD*	*M*	*SD*	*M*	*SD*
PCL-M								
Reexperiencing	8.00	4.16	8.60	5.21	7.63	5.07	10.00	5.34
Avoidance	13.40	5.97	12.80	4.73	11.50	4.17	15.00	4.53
Hyperarousal	11.00	4.69	11.10	5.80	10.63	6.44	13.80	6.61
Total	32.40	13.34	32.50	15.01	29.75	15.24	38.80	15.12
MASQ								
GDA	15.90	3.98	17.30	4.11	19.25	6.14	22.40	4.16
AA	21.30	3.65	20.60	2.59	22.25	6.25	25.80	7.05
GDD	21.60	7.34	19.40	4.79	20.13	6.81	24.20	6.53
AD	66.10	12.68	64.10	13.07	62.63	12.37	68.00	12.39
Total	124.90	17.51	121.40	19.40	124.25	22.41	140.40	18.37
Startle								
Light	32.26	36.80	29.24	27.16				
Dark	35.08	32.02	31.46	27.46				
Respiration	10.03	2.05	11.16	2.23				

	Time Active							
	1 (*n* = 11)		2 (*n* = 10)		3 (*n* = 8)		4 (*n* = 9)	
Measure	*M*	*SD*	*M*	*SD*	*M*	*SD*	*M*	*SD*
PCL-M								
Reexperiencing	9.27	3.23	7.70	2.26	8.88	3.72	7.11	1.69
Avoidance	14.45	6.52	10.10	3.14	10.50	4.41	10.22	3.83
Hyperarousal	12.82	4.71	8.20	2.53	9.25	3.73	8.22	2.59
Total	36.55	11.44	26.00	6.91	28.63	10.68	25.56	6.58
MASQ								
GDA	19.64	5.50	16.00	4.27	16.00	4.41	18.44	6.41
AA	27.00	7.84	22.70	4.74	20.75	2.49	21.67	6.40
GDD	23.00	8.04	18.10	5.17	17.38	5.37	18.78	7.82
AD	59.64	17.23	44.60	15.36	49.00	12.62	47.11	17.60
Total	129.27	32.47	101.40	26.60	103.13	22.65	106.00	35.96
Startle								
Light	82.04	104.45	61.37	68.95				
Dark	94.40	103.08	66.11	65.12				
Respiration^a^	10.20	2.12	9.07	2.81				

*Note*. PCL-M = Posttraumatic Stress Disorder Checklist-Military Version; MASQ = Mood and Anxiety Symptoms Questionnaire; GDA = General Distress-Anxiety; AA = Anxious Arousal; GDD = General Distress-Depressive; AD = Anhedonic Depression.

a Time 1 has *n* = 10 participants due to technical difficulties.

We found a Group × Time interaction for Reexperiencing *F*(3, 45.11) = 2.89, *p* =. 045, and for Hyperarousal, *F*(3, 44.7) = 6.51, *p* <. 001, but not for Avoidance, *F*(3, 45.6) = 1.74, *p* =. 171. With regard to Reexperiencing, the active group showed fewer symptoms at Time 2, *t*(45.28) = 2.11, *p* =. 040, and Time 4, *t*(45.36) = 3.13, *p* =. 003, compared to Time 1. With regard to Hyperarousal, the active group showed fewer symptoms at Time 2, *t*(44.88) = 5.45, *p* <. 001, Time 3, *t*(45) = 4.53, *p* <. 001, and Time 4, *t*(44.93) = 5.06, *p* <. 001, compared to Time 1. We observed no significant differences from Time 1 on any of the PCL-M subscales for the control group. Medium to large between-group effect sizes were observed across primary outcome measures (see Table 2).

**Table 2:** Between-Group Effect Size Relative to Time 1

	Time					
	2 (*n* = 10)		3 (*n* = 8)		4 (*n* = 9)	
Measure	*d*	95% CI	*d*	95% CI	*d*	95% CI
PCL-M						
Reexperiencing	0.81	[−0.11, 1.67]	0.34	[−0.53, 1.19]	0.91	[−0.03, 1.77]
Avoidance	0.55	[−0.34, 1.40]	0.52	[−0.37, 1.37]	0.58	[−0.32, 1.43]
Hyperarousal	1.40	[0.39, 2.29]	1.49	[0.47, 2.39]	1.06	[0.10, 1.92]
Total	1.16	[0.20, 2.04]	0.94	[0.00, 1.80]	1.00	[0.05, 1.86]
MASQ						
GDA	1.55	[0.52, 2.46]	1.98	[0.87, 2.93]	1.30	[0.31, 2.19]
AA	0.64	[−0.27, 1.49]	1.23	[0.25, 2.11]	1.07	[0.12, 1.94]
GDD	0.40	[−0.48, 1.25]	0.51	[−0.38, 1.36]	0.43	[−0.45, 1.28]
AD	0.93	[0.00, 1.79]	0.74	[−0.17, 1.59]	0.87	[−0.06, 1.72]
Total	0.96	[0.02, 1.82]	1.11	[0.15, 1.98]	0.99	[0.05, 1.86]
Startle						
Light	0.51	[−0.38, 1.35]				
Dark	0.63	[−0.27, 1.48]				
Respiration	1.22	[0.25, 2.10]				

*Note*. CI = confidence interval; PCL-M = Posttraumatic Stress Disorder Checklist-Military Version; MASQ = Mood and Anxiety Symptoms Questionnaire; GDA = General Distress-Anxiety; AA = Anxious Arousal; GDD = General Distress-Depressive; AD = Anhedonic Depression.

Total mood and anxiety (MASQ) showed a Group × Time interaction, *F*(3, 45.6) = 4.47, *p* =. 008. The active group showed fewer symptoms at Time 2, *t*(45.63) = 4.19, *p* <. 001, Time 3, *t*(46.02) = 3.83, *p* <. 001, and Time 4, *t*(45.82) = 3.31, *p* =. 002, compared to Time 1 (Table1).

When anxiety was broken down, we found a Group × Time interaction for both anxiety subscales of General Distress-Anxiety, *F*(3, 45) = 9.47, *p* <. 001, and Anxious Arousal, *F*(3, 46.6) = 4.57, *p* =. 006, but not for the depression subscales of Anhedonic Depression, *F*(3, 45.3) = 2.65, *p* =. 060, or General Distress-Depressive, *F*(3, 46.5) = 1.62, *p* =. 198. With regard to General Distress-Anxiety, the active group showed fewer symptoms at Time 2, *t*(45.12) = 3.31, *p* =. 001, and Time 3, *t*(45.31) = 3.68, *p* <. 001, whereas the control group showed more General Distress-Anxiety at Time 3, *t*(44.76) = −3.45, *p* =. 001, and Time 4, *t*(45.31) = −3.56, *p* <. 001, compared to Time 1. With regard to anxious arousal, the active group showed fewer symptoms at Time 2, *t*(46.51) = 2.69, *p* =. 009, Time 3, *t*(47.12) = 3.76, *p* <. 001, and Time 4, *t*(46.8) = 3.16, *p* =. 002, compared to Time 1.

Regarding psychophysiological measures, a Group × Time interaction was observed for respiration rate, *F*(1, 18) = 7.93, *p* =. 011; the active group showed reduced respiration rate at Time 2 relative to Time 1. Correlational analyses revealed no associations between respiration rate and the self-report scales.

A nonsignificant effect of time was observed for startle in the combined light–dark conditions, *F*(1, 17) = 3.48, *p* =. 079, *d* = 0.59, 95% CI [−0.30, 1.44] and in the dark condition only, *F*(1, 17) = 3.84, *p* =. 067, *d* = 0.63, 95% CI [−0.27, 1.48], such that the active group showed reduced startle response. No effects, however, involving Group or Group × Time were observed for eye-blink startle (all *p*s >. 138).

The predicted association between startle and PTSD symptoms was assessed for Time 1 and for intervention effects (Time 2 − Time 1). At Time 1 across both groups, PTSD symptoms on the PTSD Checklist-Military scale did not show a correlation with overall startle response across the light and dark periods, *r* =. 14, *p* =. 574 (light: *r* =. 12, *p* =. 625; dark: *r* =. 15, *p* =. 531). The subscale most relevant, however, to physiological startle, Hyperarousal, showed a nonsignificant correlation with overall startle response across the light and dark periods (light: *r* =. 40, *p* =. 088; dark: *r* =. 41, *p* =. 079).

**Figure 2:**
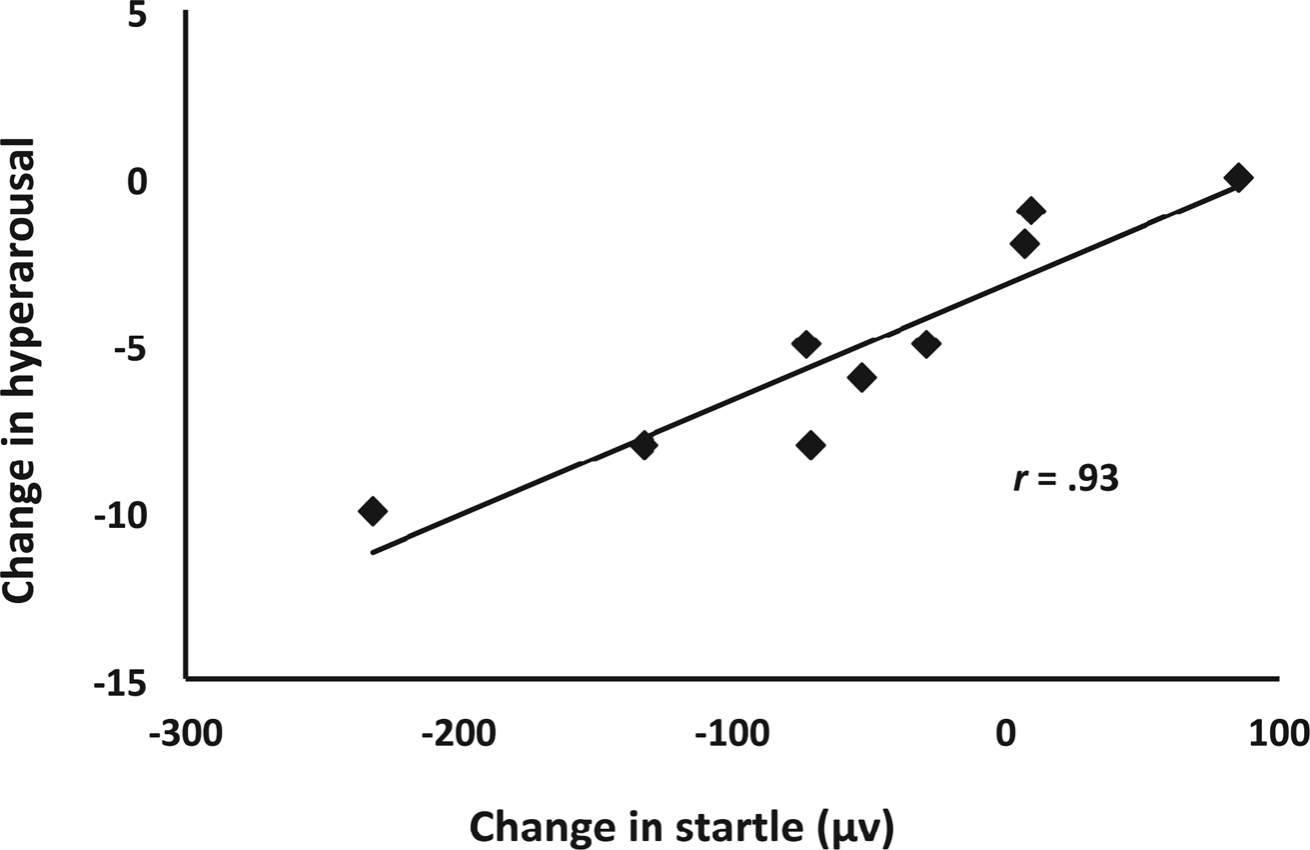
Reduction of PTSD hyperarousal symptoms following Sudarshan Kriya yoga was associated with reduction in startle response.

For intervention effects (Time 2 − Time 1), decrease in overall PTSD symptoms did not correlate significantly with startle response across the light and dark periods in the active group, *r* =. 59, *p* =. 096 (light: *r* =. 54, *p* =. 134; dark: *r* =. 60, *p* =. 085), nor in the control group. Hyperarousal, however, correlated significantly with decrease in startle response across the light and dark periods in the active group, *r* =. 93, *p* <. 001 (light: *r* =. 91, *p* =. 001; dark: *r* =. 89, *p* =. 001), but not in the control group (Figure 2). The magnitude of these correlations showed a group difference (Fisher's *z* = 2.76, *p* =. 006).

Of note, decreases in startle response due to intervention effects (Time 2 − Time 1) predicted decreases in PTSD Hyperarousal, *r* =. 77, *p* =. 025, and Reexperiencing, *r* =. 73, *p* =. 041, as well as General Distress-Anxiety, *r* =. 77, *p* =. 015, at the 1-year follow-up (Time 4 − Time 1) in the active group.

Of the veterans in the active group assessed at 1 year, seven had continued to practice Sudarshan Kriya yoga (two practiced once in the past month, two several times in the past month, one once a week, one several times a week, and one daily). No significant correlations, however, were found between amount of continued practice and self-reported changes in PTSD and anxiety.

## Discussion

This is the first randomized trial of Sudarshan Kriya yoga for Afghanistan and Iraq veterans of which we are aware. It resulted in reduced PTSD symptoms, anxiety, and respiration rate. Reduction in startle, though nonsignificant, correlated robustly with reduction in self-reported hyperarousal symptoms in the active group, providing an objective measure to substantiate the self-report measures. Ninety percent of veterans in the active group completed the study, suggesting high acceptability of Sudarshan Kriya yoga as an intervention for Afghanistan or Iraq veterans.

Sudarshan Kriya yoga showed the strongest effect on hyperarousal and reexperiencing symptoms, and consistent with improvements in hyperarousal symptoms, also on generalized anxiety and arousal symptoms. These findings are promising given that of the three PTSD symptoms, hyperarousal yields the strongest influence on health-related quality of life in returning veterans (Doctor, Zoellner, & Feeny, 2011). Furthermore, the lack of a correlation between continued practice and changes in PTSD and anxiety symptoms suggests that the benefits of the 1-week Sudarshan Kriya yoga program for PTSD and associated symptoms endured independently of continued practice.

The growing body of data demonstrating Sudarshan Kriya yoga's promise for a range of psychological disorders (Zope & Zope, 2013) raises the question of theories that may explain its success. Brown and Gerbarg (2005a) propose a theory of the neurophysiological mechanisms underlying Sudarshan Kriya yoga. Briefly, they state that by activating both the parasympathetic and sympathetic system, the breathing exercises may produce a state of both alertness and calm. Benson's relaxation response is meant to work in a similar fashion: calming the mind by relaxing the body (Chang, Dusek, & Benson, 2011). In their follow-up review, Brown and Gerbarg (2005b) provide evidence of the beneficial effects of Sudarshan Kriya yoga as an adjunct to current standard treatment in clinical populations with a wide range of symptoms (e.g., anxiety, depression, substance abuse).

Emotional processing theory (Rauch & Foa, 2006) posits that habituation through exposure can disrupt the relationship between a stimulus and a conditioned fear response. Similarly, Sudarshan Kriya yoga may reduce trauma by decoupling the stimulus (i.e., recalled memories) from the fear response: Participants reported reexperiencing traumatic memories while in a breathing-induced, relaxed, physiological state. After the intervention, they reported that these traumatic memories no longer impacted them as strongly.

Many studies have examined the benefits of mindfulness-based meditation. Fewer studies have been conducted on breathing practices. Sudarshan Kriya yoga, however, may hold distinct advantages over mindfulness-based interventions. Meta-analytic reviews of research on mindfulness indicate that its impact on anxiety is often low, ambiguous, or equivalent to a control intervention (Goyal et al., 2014; Toneatto & Nguyen, 2007). Since sitting silently in meditation – as in traditional mindfulness practices – may be challenging for anxious people with high degrees of physiological arousal, breathing may be preferable because it engages the participant in a structured activity (i.e., controlled breathing) that leads to immediately observable calming effects (Brown & Gerbarg, 2005b). Given that changes in respiration can alter emotional states (Boiten et al., 1994; Philippot et al., 2002), manipulation of the breath may offer control over anxiety, further underscoring the applicability of respiration-based interventions for anxious populations (Arch & Craske, 2006; Asmundson & Stein, 1994; Kaushik et al., 2006; Salkovskis et al., 1986).

Limitations include a small sample size lacking demographic representation. Future research should assess generalizability across genders, ethnicities, military cohorts, and PTSD symptom severity, and include active control groups to test for the impact of group cohesion (MacCoon et al., 2012). A formal assessment of comorbidities would help identify specificity of symptom changes. Mechanistically oriented studies could help determine how Sudarshan Kriya yoga benefits trauma (e.g., through changes in brain activation).

Despite these limitations, the study includes numerous methodological strengths often absent in studies of novel interventions: a waitlist control group, randomization, intention-to-treat analyses, range of outcome variables (self-report, psychophysiological), and two follow-up assessments (1 month and 1 year).

Given the debilitating impact of PTSD on returning veterans and the limited success of current interventions in this population, there is a need to expand the range of intervention options available. This study found that a breathing-based meditation intervention resulted in improvements on psychophysiological and symptom measures. Sudarshan Kriya yoga, a week-long intervention with longitudinal benefits, shows promise as a viable alternative or adjunct intervention for addressing PTSD and suicide in returning veterans.
